# Annual Meeting to Award Grants

**Published:** 1914-12-26

**Authors:** 


### KING EDWARD'S HOSPITAL FUND FOR LONDON.

### Annual Meeting to Award Grants.

A meeting of the Governors and General Council of King Edward's Hospital Fund for London for the purpose of awarding grants to the hospitals, convalescent homes, and consumption sanatoria for the present year, was held on Wednesday morning, the 16th inst., at St. James's Palace, the Speaker of the House of Commons being in the chair.

### The Treasurer's Statement.

In the absence of Lord Rothschild (the honorary treasurer), Lord Revelstoke said that the amount received to the 12th instant was: On capital account, £370,856 10s. 11d.; and on general account, after payment of expenses, £133,706 13s. 3d.

The receipts on capital account were made up chiefly of the first instalments of the legacy of Sir Julius Wernher, which was left to capital, together with £30,863 further from the Lewis estate, and the Harman bequest of ground rents, valued at £25,000, which brought in £1,382 a year. From the Wernher estate the Fund had received so far a sum of £313,715.

With regard to the receipts on general account, it was too early to say anything about the effects of the war, except that up to the present there had been considerably less reduction in annual subscriptions, donations, and income from investments than the executive committee, as a matter of prudence, had allowed for at the time when the amount for distribution was fixed. There was, however, a slight reduction; and special efforts would no doubt have to be made next year if the inevitable loss of support from those who were seriously affected by the war was to be made up.

An Increase in the League of Mercy's Collections.

Sir Henry Burdett, in making his annual statement on behalf of the League of Mercy, said that in the peculiar and special circumstances of the present year it was remarkable and satisfactory to be able to state that the reports of the presidents, lady presidents, and their workers of the League of Mercy showed that the total sum raised in 1914 exceeded the amount received in 1913. It was too early to fix the actual sum which it would be possible to hand over to the treasurer of the King's Fund; but it was a very real pleasure to be able to make so satisfactory a report to the Council in a year like the present.

### The King's Letter.

The Speaker of the House of Commons, in moving the adoption of the reports and awards of the Distribution and Convalescent Homes Committee [see pp. 291-293], said:

My Lords and Gentlemen,—As the Duke of Teck, whose whole time is taken up with his military duties, is unable to be present, I rise on behalf of the Governors to move the adoption of the reports. His Majesty has been graciously pleased to write a letter, through Lord Stamfordham, which I think at this point I ought to read:

"Buckingham Palace.

"Dear Sir,—I am commanded by the King to ask you to convey to the Council the expression of his continued interest in the Fund.

"His Majesty appreciates the reasons which have led the Council to fix the distribution for this abnormal year, under conditions wholly unprecedented, at £140,000.

"The supporters of the Fund will note with satisfaction the fact that the hospitals have for the time being wisely limited their capital liabilities, and that the Council has, therefore, been enabled to increase the total grants made for maintenance purposes.

"While the general public is giving ample evidence of ready sacrifice for national objects, the King trusts that the pressing needs of the voluntary hospitals during the present crisis will not be lost sight of, and that the Fund may receive such support as to be able to increase its annual distribution next year.

"His Majesty has observed with lively interest the fact that the professional staffs of the London hospitals have been largely called upon to assist, at the seat of the war as well as at home, in treating the sick and wounded. Many contingents from the nursing staffs are working abroad in their professional capacities, while hospitals in London have in no small measure assisted in dealing with the wounded brought to England.

"The King learnt with regret during the year now drawing to its close that the Fund had received the resignation, through ill-health, of Mr. Latham, who for many years had been Chairman of the Convalescent Homes Committee.

"His Majesty desires to record his grateful testimony to the valuable services rendered by all who have assisted in dealing with the work of the Fund, and especially recognises the ready manner in which Mr. Fry kindly undertook to carry on the duties of Sir Savile Crossley during the absence of the latter at the seat of war.

"Yours very faithfully,
"Stamfordham.

"The Presiding Governor,
"King Edward's Hospital Fund for London"

### The Speaker's Speech.

The point which I wish first to emphasise is the fact that we are increasing the maintenance grants to the hospitals. As the Council are well aware, the question of the amount to be distributed required special consideration this year, in view of the probable effect of the war both on the hospitals and on the Fund. The evidence available at the time the amount was settled pointed to the conclusion that in both cases the effect would probably be more marked later on than it would this year. If, therefore, we had made a special effort to maintain the distribution at £157,500 (the figure since 1911), there would have been some risk of a reduction next year, just when the needs of the hospital might be expected to be increasing. Moreover, the demands on the Fund for grants in aid of capital expenditure are less this year than usual, owing to the postponement of several schemes of building or improvements. A distribution of the same amount as last year would, therefore, have meant large additional grants to maintenance, and it is not yet possible to know with accuracy which hospitals will be affected most seriously by the war, and will therefore most need exceptional assistance.

An Essential Form of National Service.

There can be no doubt that a great effort will be necessary if the work of the hospitals is to be maintained during the time of stress that is already upon them. Happily, there is in the nation a universal desire to be of use and to help the country in its emergency. To large numbers of people the giving of money to public objects is one of the readiest methods of helping. The various great war relief funds form one outlet for this feeling; but another and scarcely less important channel is the support of the voluntary hospitals. For surely their work in combating disease and maintaining the health of the people—to say nothing of their direct assistance to the naval and military authorities in the treatment of sick and wounded sailors and soldiers—is in itself one of the most essential forms of national service.

The very welcome announcement made by Sir Henry Burdett of the large sums collected for the Fund this year by the League of Mercy is evidence that where this is recognised an immediate response is forthcoming.

If the needs of the hospitals are once realised, it must also be recognised that the financial assistance of the Fund is one of the great factors in their support. The Fund has exceptional facilities for ascertaining at which hospitals the greatest need of assistance exists, and allocates its grants accordingly, provided, of course, that due regard is paid to economy and efficiency. If, however, the Fund is to make full use of its opportunities, it is essential, not only that the amount of the distribution should be maintained, but that it should be increased as the difficulties of the hospitals increase. And for this we depend on the support of the charitable public.

In consequence of the great national emergency, the ordinary activities of the Fund have naturally fallen somewhat into the background. But there are a few points which I should like to mention.

The question of pensions for hospital officers has been more than once brought to the notice of the Fund as a matter that called for consideration, particularly in the case of the smaller hospitals. Early in the year the Executive Committee appointed a sub-committee to inquire into the subject. This sub-committee, under the expert chairmanship of Mr. W. J. H. Whittall, had, at the end of the summer, contemplated the preparation of a preliminary report which might help to ventilate the subject and perhaps lead to a general agreement. Owing to the outbreak of the war the actual preparation of this preliminary report has been delayed; but it is still under consideration.

### The Revised Uniform System.

The Revised Uniform System of Hospital Accounts has now reached what may prove to be a final form (in so far as finality is possible in such matters) by the publication of a new edition, embodying all the decisions on points of difficulty that have arisen since the original issue. The application of the system to the circumstances of each institution has meant a good deal of work on the part of the hospital officials and also of their auditors, to whom I think a special word of thanks is due. Quite apart from the labour involved, it is no light thing for a professional expert, who has himself perhaps introduced a perfectly sound system adapted to the needs of a particular institution, to be asked to abandon it in favour of a different system coming from outside. But this was a sacrifice which the three Funds were compelled to ask, for the sake of the degree of uniformity necessary to a fair distribution, and we are grateful to the hospital auditors for the willingness and public spirit with which they complied.

### Sir Julius Wernher's Munificent Legacy.

One important event of the year, which certainly does not fall into the background, is the receipt of four instalments of the munificent legacy from the late Sir Julius Wernher, given expressly in augmentation of the endowment of the Fund. We have received so far a sum of £313,000 on account of this bequest. On behalf of the Governors and of this Council I should like to express our thanks to Lady Wernher and the other executors of the will for the constant consideration and care for the interests of the Fund which they have shown in the administration of the estate.

To come to matters personal to the Fund, we very much regret that ill-health has deprived us of the services of Mr. William Latham, who had for sixteen years served the Fund with equal skill and tact as Chairman of the Convalescent Homes Committee. In all that period he only missed one meeting of the committee. We hope he will still feel able to retain his membership of the Council.

The Convalescent Homes Committee has also lost by death the services of Mr. Benson, who had previously been a member of the Organising Committee of the Coronation gift in 1902, and of the Executive Committee, and a visitor. We have also lost a very useful visitor by the death of Mr. Reginald Lucas. To the lamented death of Lord Strathcona, one of our greatest benefactors, as well as an active member of the Council, reference was made at the annual meeting in April last.

Besides the Duke of Teck, several members of the Council, members of committees and visitors of the Fund, both medical and lay, are engaged on special duties connected with the war, many of them being at the seat of war. During their absence Mr. Fry came forward with his usual readiness to the assistance of Mr. Griffiths, who continues to give to the work of the Fund a great deal of time which he can only with difficulty spare from other numerous and responsible engagements.

I should also like to refer to one point which Lord Revelstoke has not mentioned in his statement just now —namely, the very valuable services which Messrs. Barings have, with their usual courtesy and generosity, rendered during the crisis, in connection with the funds awaiting distribution.

I have now only to express the thanks of the Governors to the Distribution and Convalescent Homes Committees for their reports, and to the visitors whose work helps us to keep in personal touch with the hospitals every year.

Sir Henry Burdett, in supporting the motion for the adoption of the Reports, referred to the great services rendered by Mr. Griffiths in connection with the Revised Uniform System of Hospital Accounts. The amount of time and work involved was far greater than anyone not intimately associated with matters of that kind could realise, and the warmest thanks of the Council were due to Mr. Griffiths.

On the motion of Sir William Church (Chairman of the Distribution Committee), seconded by Sir Cooper Perry, a report of the committee upon a communication from the Westminster Hospital with reference to their proposals for removal was adopted.

After the announcement of the appointment of members of the Council and committees and of honorary officers for 1915, the approval of resolutions delegating powers to the various committees and the moving of a vote of thanks to the Speaker, the proceedings terminated.

